# Azole Resistance-Associated Regulatory Motifs within the Promoter of *cyp51A* in Aspergillus fumigatus

**DOI:** 10.1128/spectrum.01209-22

**Published:** 2022-05-16

**Authors:** Alexander Kühbacher, Mandy Peiffer, Peter Hortschansky, Petra Merschak, Michael J. Bromley, Hubertus Haas, Axel A. Brakhage, Fabio Gsaller

**Affiliations:** a Institute of Molecular Biology, Biocenter, Medical University of Innsbruck, Innsbruck, Austria; b Department of Molecular and Applied Microbiology, Leibniz Institute for Natural Product Research and Infection Biology, Jena, Germany; c Manchester Fungal Infection Group, Division of Infection, Immunity, and Respiratory Medicine, The University of Manchester, Manchester, United Kingdom; d Institute of Microbiology, Friedrich Schiller University Jena, Jena, Germany; Universidade de Sao Paulo

**Keywords:** *Aspergillus fumigatus*, regulatory elements, iron regulation, transcription factors, *cyp51A*, azole resistance

## Abstract

Aspergillus fumigatus is one of the deadliest fungal species, causing hundreds of thousands of deaths each year. Because azoles provide the preferred first-line option for treatment of aspergillosis, the increase in rates of resistance and the poor therapeutic outcomes for patients infected with a resistant isolate constitute a serious global health threat. Azole resistance is frequently associated with specific tandem repeat duplications of a promoter element upstream of *cyp51A*, the gene that encodes the target for this drug class in A. fumigatus. This promoter element is recognized by the activating transcription factors SrbA and AtrR. This region also provides a docking platform for the CCAAT-binding complex (CBC) and HapX, which cooperate in the regulation of genes involved in iron-consuming pathways, including *cyp51A*. Here, we studied the regulatory contributions of SrbA, AtrR, CBC, and HapX binding sites to *cyp51A* expression and azole resistance under different iron availability employing promoter mutational analysis and protein-DNA interaction analysis. This strategy revealed iron status-dependent and -independent roles of these regulatory elements. We show that promoter occupation by both AtrR and SrbA is required for iron-independent steady-state transcriptional activation of *cyp51A* and its induction during short-term iron exposure relies on HapX binding. We further reveal the HapX binding site as a repressor element, disruption of which increases *cyp51A* expression and azole resistance regardless of iron availability.

**IMPORTANCE** First-line treatment of aspergillosis typically involves the use of azole antifungals. Worryingly, their future clinical use is challenged by an alarming increase in resistance. Therapeutic outcomes for such patients are poor due to delays in switching to alternative treatments and reduced efficacy of salvage therapeutics. Our lack of understanding of the molecular mechanisms that underpin resistance hampers our ability to develop novel therapeutic interventions. In this work, we dissect the regulatory motifs associated with azole resistance in the promoter of the gene that encodes the azole drug target Cyp51A. These motifs include binding platforms for SrbA and AtrR, as well as the CCAAT-binding complex and HapX. Employing mutational analyses, we uncovered crucial *cyp51A*-activating and -repressing functions of the binding sites. Remarkably, disrupting binding of the iron regulator HapX increased *cyp51A* expression and azole resistance in an iron-independent manner.

## OBSERVATION

The human mold pathogen Aspergillus fumigatus is the most common cause of invasive aspergillosis. Azole antifungals constitute the major drug class employed for first-line treatment of this life-threatening disease (1). Over the past years, a worrying increase in resistance to this drug class has been reported, and the U.S. Centers for Disease Control and Prevention have recently added azole-resistant A. fumigatus to their watch list for antibiotic resistance threats (2).

**FIG 1:**
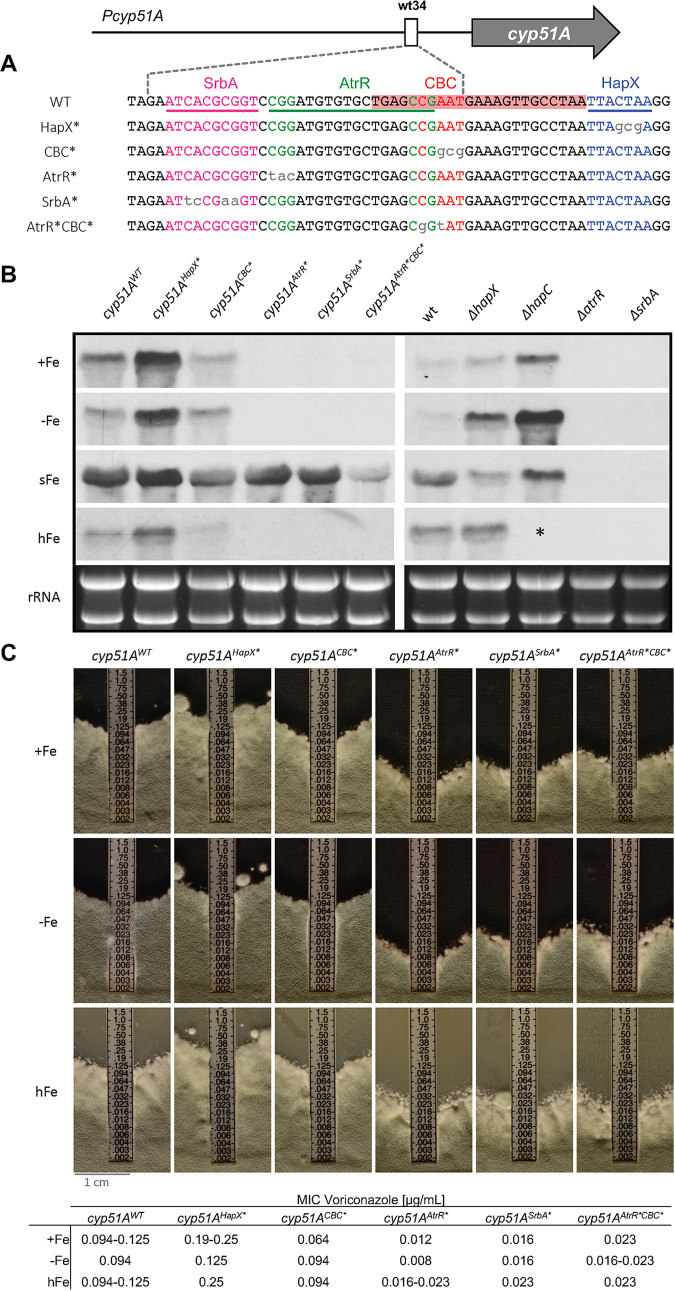
Analysis of *cyp51A* expression and voriconazole susceptibility testing of *Pcyp51A* mutants. (A) To disrupt interaction of AtrR, SrbA, the CBC, and HapX at the 34-mer, specific base pair changes (gray lowercase letters) were introduced into their binding motifs. The binding sites of SrbA, AtrR, and HapX are underlined, and the DNA area covered by the CBC is highlighted in red. (B) *cyp51A* transcript levels were determined in mutants containing *cyp51A* expression constructs under the control of differently mutated *Pcyp51A*, as well as the corresponding transcription factor loss-of-function mutants. (C) Voriconazole susceptibility of *Pcyp51A* mutants was analyzed using Etest. *, Δ*hapC* was not able to grow under hFe.

Azoles target the ergosterol biosynthesis enzyme Cyp51, which catalyzes the 14α-demethylation of eburicol in A. fumigatus (3). Cyp51 inhibition decreases ergosterol production and causes eburicol accumulation, which in turn leads to increased production of toxic C14-methylated sterols (4). Although A. fumigatus expresses two Cyp51 isoenzymes (Cyp51A and Cyp51B), azole resistance mechanisms are rarely linked to mutations of *cyp51B* (5, 6). Two predominant mechanisms of resistance, designated TR34/L98H and TR46/Y121F/T298A, both involve a tandem repeat (TR) within the promoter of *cyp51A* (*Pcyp51A*) coupled with mutations in the coding sequence, leading to amino acid alterations in the enzyme (7, 8). *In vitro* studies using recombinant strains transformed with *cyp51A* mutant alleles demonstrated that the TRs elevate *cyp51A* expression, which results in increased azole resistance (9, 10). To date, several TR variants have been discovered in azole-resistant clinical isolates, including the aforementioned TR34 and TR46 as well as TR53 and TR120 (7, 8, 11, 12). It is important to note that the 34-mer (wt34) duplicated in TR34 is also present in all of the other TRs (12, 13), suggesting that this DNA region is a key element driving azole resistance. Previous work suggested that wt34, displayed in Fig. 1A, contains binding sites for the *cyp51A*-activating factors SrbA and AtrR (13–15). Overlapping the AtrR binding site, wt34 harbors an imperfect CCAAT box (5′-CGAAT-3′) that is recognized by the CCAAT-binding complex (CBC) (13, 16). A HapX binding motif is located in close proximity to the CBC (13). Depending on the cellular iron status, the CBC and HapX cooperatively (CBC-HapX) activate or repress the expression of genes involved in iron-dependent pathways such as *cyp51A* (17–19).

In addition to *cyp51A*, AtrR, SrbA, and CBC-HapX were shown to directly regulate the expression of numerous other target genes, including those coding for metabolic enzymes and transcriptional regulators (13–15, 20, 21). Therefore, it is likely that their inactivation affects various cellular processes, and the possibility that changes in individual gene expression, including that of *cyp51A*, are based on indirect effects cannot be excluded. In this study, we generated *Pcyp51A* mutants harboring disrupted binding motifs for SrbA, AtrR, the CBC, and HapX to study their direct contributions to *cyp51A* regulation and azole resistance. Due to the involvement of these regulators in the control of iron metabolism (14, 20, 22, 23), we monitored the effects of disrupted binding sites on *cyp51A* expression under different iron abundance.

**FIG 2:**
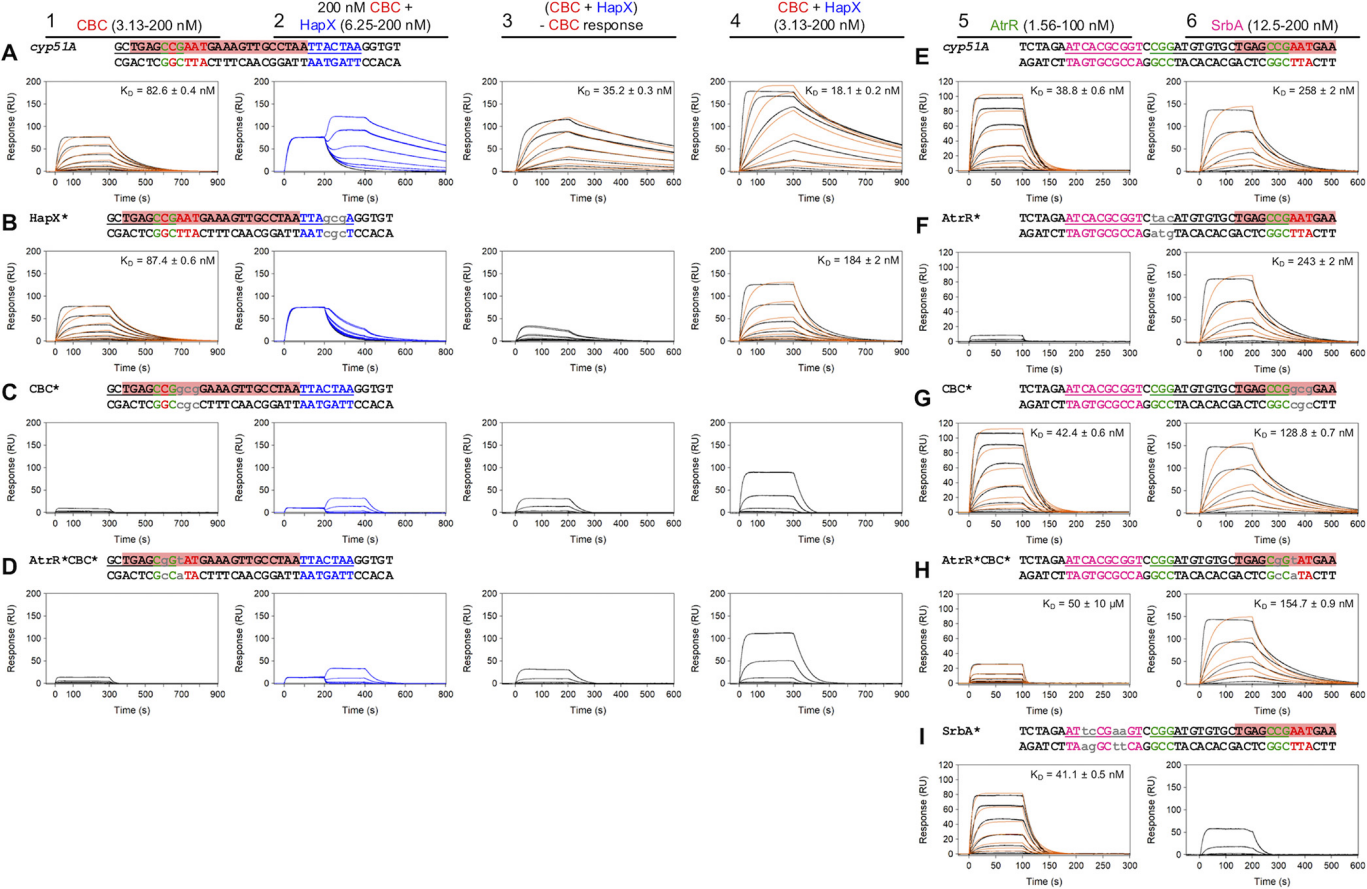
SPR-based analysis of SrbA, AtrR, CBC, and HapX binding to their consensus binding motifs at the 34-mer. Sensorgrams for binding of CBC to DNA (panel 1), HapX to preformed CBC-DNA complexes (panel 2), premixed CBC and HapX to DNA (panel 4), AtrR to DNA (panel 5), and SrbA to DNA (panel 6) are shown. Sensorgrams in panel 3 show the association/dissociation responses of HapX on preformed CBC-DNA. The CBC response was subtracted (coinjection of buffer) from HapX coinjection responses. Interactions with nonmutated (A and E) and mutated (B to D and F to I) binding sites were monitored. Binding responses of the indicated SrbA, AtrR, CBC, or HapX concentrations injected in duplicate (black lines) are overlaid with the best fit derived from a 1:1 interaction model, including a mass transport term (red lines). Binding responses of CBC-DNA-HapX ternary complex formation (panel 2, blue lines) were obtained by concentration-dependent coinjection of HapX on preformed binary CBC-DNA complexes after 200 s in the steady-state phase. Dissociation constant (*K_D_*) values of the complexes are displayed inside the graphs.

Prior to *in vivo* expression analyses, the impact of the introduced mutations on the binding affinity of these transcription factors was analyzed *in vitro* using surface plasmon resonance (SPR) protein-DNA interaction analysis. The binding affinity of SrbA and AtrR was severely affected by the introduced mutations in their specific predicted binding sites (Fig. 2F and H, panel 5 for AtrR and Fig. 2I, panel 6 for SrbA) but was largely unaffected by mutations in the adjacent putative binding motifs for the other transcription factors (Fig. 2G and I, panel 5 for AtrR and Fig. 2F, G and H, panel 6 for SrbA). This is particularly important to discriminate binding of the CBC and AtrR, because their binding motifs overlap (Fig. 1A). Comparing DNA binding affinities of the CBC (Fig. 2A, panel 1) with that of premixed CBC and HapX (Fig. 2A, panel 4) and HapX with preformed CBC-DNA (Fig. 2A, panel 2 and 3) indicated that HapX improves binding of the CBC to DNA. This was true even for its interaction at the mutated CBC binding motif, which was not recognized by the CBC alone (Fig. 2C and D, panel 1 and 4). The observed assistance of HapX for CBC-DNA binding might be due to the fact that the CBC recognition motif is an imperfect CCAAT box with only moderate CBC affinity (20). Mutation of the predicted HapX binding site impaired binding of HapX to preformed CBC-DNA complexes (Fig. 2B, panel 2 and 3), as well as that of premixed CBC and HapX (Fig. 2B, panel 4 ), which is consistent with the idea that HapX assists CBC-DNA interaction at wt34.

For *in vivo* expression analyses, *cyp51A* gene cassettes containing nonmutated and mutated *Pcyp51A* variants (Fig. 1A), analogous to those assessed by SPR (Fig. 2), were integrated at its native locus (see Fig. S1 in the supplemental material). Differential expression resulting from disrupted binding sites was determined by comparing *cyp51A* transcript levels of *Pcyp51A* mutants (cyp51A^AtrR^*, cyp51A^SrbA^*, cyp51A^CBC^*, cyp51A^HapX^*, and cyp51A^AtrR^*^CBC^*) to those of the strain carrying the nonmutated promoter (*cyp51A^WT^*). To identify potential iron status-specific effects, *cyp51A* expression was monitored during iron starvation (0 mM FeSO_4_, −Fe), sufficiency (0.03 mM FeSO_4_, +Fe), and excess (5 mM FeSO_4_, hFe) and during short-term iron adaptation via supplementation of −Fe cultures for 30 min with iron (sFe). Furthermore, the regulation patterns of *Pcyp51A* mutants were compared to those of the respective transcription factor loss-of-function mutants.

HapX, together with the CBC, was previously shown to mediate repression of *cyp51A* during iron starvation and upregulation during short-term iron exposure (18). This is in accordance with the *cyp51A* transcript levels found in wild-type (wt) and mutant strains lacking HapX (Δ*hapX*) or CBC (Δ*hapC*), as well as in the strains carrying the unmutated (*cyp51A^WT^*) and mutated (cyp51A^HapX^*) HapX binding motif (Fig. 1B); importantly, sFe-mediated induction is characterized by increased transcript levels during sFe, compared to −Fe. Interestingly, in contrast to HapX inactivation (Δ*hapX*), mutation of its binding site (cyp51A^HapX^*) caused increased *cyp51A* expression also under +Fe and hFe. Remarkably, mutation of the CBC binding site (cyp51A^CBC^*) resulted in lower *cyp51A* transcript levels, compared to mutation of the HapX binding site (cyp51A^HapX^*), and did not fully abolish *cyp51A* induction during sFe (Fig. 1B). We hypothesize that HapX dominates the sFe induction of *cyp51A* and DNA binding of the CBC plays a minor role for iron regulation in this case. In the cyp51A^CBC^* mutant, the remaining repressing (−Fe and +Fe) and inducing (sFe) capacities of the CBC could be related to the potential stabilizing properties of HapX found for *in vitro* binding of the CBC to its mutated binding motif (Fig. 2C and D, panel 1 and 4). The different expression patterns observed for the cyp51A^HapX^* and cyp51A^CBC^* mutants, compared to those of their corresponding loss-of-function mutants, could be a result of indirect regulatory defects or the potential stabilization of the CBC or HapX by other proteins at the 34-mer.

Mutation of either the AtrR or SrbA binding motif (cyp51A^AtrR^* and cyp51A^SrbA^*), as well as loss of the corresponding transcription factors (Δ*atrR* and Δ*srbA*), led to severely decreased *cyp51A* expression under −Fe, +Fe, and hFe (Fig. 1B), which agrees with the *cyp51A*-activating roles of AtrR and SrbA. These findings suggest that the AtrR and SrbA motifs analyzed here are the physiologically most relevant *in vivo* binding sites despite the presence of other predicted SrbA binding motifs within *Pcyp51A* (13, 22, 24). The results further emphasize the interdependency of these two transcription factors, as previously shown for a subset of common target genes (14, 25), and demonstrate for first time that both SrbA and AtrR have to bind to wt34 in *Pcyp51A* for steady-state transcriptional activation. Surprisingly, in contrast to Δ*srbA* and Δ*atrR*, the cyp51A^SrbA^* and cyp51A^AtrR^* mutants were still able to fully induce *cyp51A* expression during sFe. These results demonstrate that AtrR and SrbA are both required for sFe induction but not their binding to wt34. SrbA was previously shown to be required for transcriptional activation of HapX (22). Consequently, SrbA and AtrR might be indirectly required for sFe induction, which is presumably exclusively mediated by the CBC-HapX complex.

The mutation impairing binding of both AtrR and CBC by affecting the overlapping consensus motifs (cyp51A^AtrR^*^CBC^*) (Fig. 1A) displayed a combination of the defects caused by the individual disruptions of AtrR (cyp51A^AtrR^*) and CBC (cyp51A^CBC^*) binding. As observed for the cyp51A^AtrR^* mutant, which lacks AtrR-mediated steady-state activation of *cyp51A*, *cyp51A* transcript levels were absent in the cyp51A^AtrR^*^CBC^* mutant under −Fe, +Fe, and hFe. During sFe, *cyp51A* induction was diminished in cyp51A^AtrR^*^CBC^*, which could be related to the reduced inducing capacity that has been detected in the cyp51A^CBC^* mutant.

In the next step, we monitored the impact of disrupted binding motifs on iron-dependent voriconazole susceptibility using Etest (bioMérieux). Mutants with defective steady-state *cyp51A* activation (cyp51A^AtrR^*, cyp51A^SrbA^*, and cyp51A^AtrR^*^CBC^*) showed increased susceptibility under −Fe, +Fe, and hFe (Fig. 1C). These data demonstrate that defective binding of either AtrR or SrbA to *Pcyp51A* at wt34 causes dramatically increased azole susceptibility. The resistance pattern of the cyp51A^CBC^* mutant was similar to that of the *cyp51A^WT^* strain under −Fe and hFe. In agreement with reduced *cyp51A* expression in the cyp51A^CBC^* mutant, this strain was slightly more susceptible under +Fe. The cyp51A^HapX^* mutant, showing increased steady-state *cyp51A* transcript levels during different iron abundance, displayed elevated resistance independent of the iron status. Similar results were obtained for voriconazole, posaconazole, and itraconazole using the European Committee on Antimicrobial Susceptibility Testing (EUCAST) broth microdilution reference method (see Table S3 in the supplemental material) (26).

TR-based *cyp51A* overexpression represents a major cause driving azole resistance in A. fumigatus (7, 8, 11, 12). Hence, a profound understanding of regulatory mechanisms associated with TRs is crucial to elucidate therapeutic strategies that counteract resistance. In this work, we explored iron status-dependent, regulatory functions of AtrR, SrbA, CBC, and HapX binding motifs located at the azole resistance-associated 34-mer, the key enhancer element involved in TR-based *cyp51A* upregulation (13). We identified iron-independent, *cyp51A*-activating functions of AtrR and SrbA binding sites during steady-state cultivation and found the HapX binding motif to be crucial for induction of the gene during short-term iron exposure.

Despite the known −Fe-specific repressing role of HapX on iron-consuming genes such as *cyp51A* (17, 18, 23), our results suggest a requirement for its functional binding motif for the repression of *cyp51A* during steady-state growth, regardless of the iron availability. Hampering this repression by disrupting the HapX binding site elevates azole resistance and thus reveals its binding motif as a potential azole resistance hot spot.

Mutation of either the SrbA binding site or the AtrR binding site within wt34 blocked *cyp51A* activation, which demonstrates that the functionality of only one is insufficient for *cyp51A* expression. The interdependency of AtrR and SrbA in *cyp51A* activation and azole resistance most likely explains why the common region (wt34) of TRs from resistant clinical isolates harbors duplicated binding sites for the both regulators (12, 13).

For details on the materials and methods, see the supplemental material.
