# Supplementary material for: Azole Resistance-Associated Regulatory Motifs within the Promoter of cyp51A in Aspergillus fumigatus
Source: Microbiol Spectr. 2022 May 16;10(3):e01209-22. doi: 10.1128/spectrum.01209-22 (PMC9241776; doi:10.1128/spectrum.01209-22)
Supplement: SUPPLEMENTAL FILE 1 — Supplemental material. Download spectrum.01209-22-s001.pdf, PDF file, 0.3 MB [file spectrum.01209-22-s001.pdf]

Supplemental Information

Materials and Methods

Strains, media and growth conditions

Strains used in this study are listed in Table S1. The strain A1160P+ (1) was used as parental strain and is referred to as wild-type (wt). Spores were generated on Sabouraud Dextrose Bouillon-Medium (SAB; Sigma-Aldrich, St. Louis, Missouri, USA) for 72 h and harvested in 10 mL spore buffer (0.9 % (w/v) NaCl; 0.1 % (v/v) Tween 20). *Aspergillus* minimal medium (AMM) containing 1 % glucose as carbon source and 20 mM ammonium tartrate as nitrogen source was used in all experiments. All incubations were performed at 37 °C.

Generation of Pcyp51A mutants

Oligonucleotides used for generation of plasmids are listed in Table S2. First, a Δ*cyp51A* strain was generated which served as recipient strain for all *cyp51A* expression constructs comprising non-mutated as well as differently mutated *Pcyp51A* variants. Therefore, a deletion fragment containing 5' and 3' non-translated regions (NTRs) connected to a zeocin resistance cassette (Fig. S1) was amplified using FusionPCR as previously described and transformed into wt (1). Second, the plasmid pcyp51A^{WT} comprising non-mutated *Pcyp51A* was generated. For this, *cyp51A* coding sequence plus approximately 1.5 kb and 2.0 kb of the 5' and 3' NTRs was amplified from genomic DNA using cyp51A-FW/RV. In addition, a plasmid backbone containing a hygromycin resistance cassette was amplified with pAN7.1-cyp51A-FW/RV using the phapC^{GFP} plasmid (2) as template. Both fragments were assembled using NEBuilder (New England Biolabs Inc., Ipswich, MA, USA). Mutation of specific transcription factor binding sites were introduced into pcyp51A^{WT} using the QuikChange Site-directed mutagenesis kit (Agilent, Santa Clara, California, USA). For the disruption of the HapX, CBC, AtrR, SrbA as well as the AtrR/CBC-overlapping region primers HapX*-FW/RV, CBC*-FW/RV, AtrR*-FW/RV, SrbA*-FW/RV and AtrR*CBC*-FW/RV were employed.

Fungal transformation

Protoplasts were transformed with 1 µg of PCR product or *Kpn*I linearized plasmid as previously described (3). Selection of positive clones was performed on Yeast-Peptone-Glucose-Sucrose-medium (YPGS; 2 % (w/v) yeast extract, 0.5 % (w/v) peptone, 2 % (w/v) D-glucose) containing 1 M sucrose for osmotic stability. Depending on the resistance cassette, media were supplemented with 150 µg/mL zeocin (Invivogen, San Diego, California, USA) or 200 µg/mL hygromycin B (Thermo Fisher Scientific, Waltham, Massachusetts, USA). Selection using zeocin and hygromycin B was carried out at pH 8 and pH 6, respectively.

Antifungal activity assay

E-tests (Biomerieux®) with voriconazole were performed according to the manufacturer's protocol (bioMérieux, Marcy-l'Étoile, France) on solid AMM. In brief, harvested conidia were diluted to 10^6/mL and distributed on the plates using a cotton swab. MIC values were obtained after 48 h of incubation where the inhibition zone intersects the strip. In addition to E-test-based susceptibility testing, voriconazole, posaconazole and itraconazole MIC levels were determined using the EUCAST (European Committee on Antimicrobial Susceptibility Testing) broth microdilution reference method (4).

Both assays were carried out under iron starvation (-Fe: 100 µM bathophenanthroline disulfonate (BPS)), iron sufficiency (+Fe: 0.03 mM $FeSO_4$) as well as iron excess (hFe: 10 mM $FeSO_4$). The strain *cyp51A*^{WT} served as reference for azole susceptibility.

Gene expression analysis

For gene expression analysis, cultures were grown for 18 h in 100 mL liquid AMM (10^6/mL) with (+Fe: 0.03 mM $FeSO_4$; hFe: 5 mM $FeSO_4$) or without iron supplementation (-Fe: 0 mM $FeSO_4$). To create short-term iron excess (sFe), cultures were incubated for 18 h under -Fe conditions before adding 0.03 mM $FeSO_4$ for 30 min. Total RNA extraction with TRI Reagent (Sigma-Aldrich, St. Louis, Missouri, USA) was performed according to the manufacturer's protocol and Northern analysis was carried out as described previously (5).

Purification of recombinant proteins and SPR based binding analysis

The *A. fumigatus* CBC consisting of the conserved core domains of $HapB_{230-299}$, $HapC_{40-137}$ and $HapE_{47-164}$ as well as $HapX_{24-158}$ were produced and purified as described and real-time SPR protein-DNA interaction measurements of the CBC as well as cooperative CBC-HapX DNA binding analysis were performed according to previously published protocols (2, 6).

Synthetic genes coding for the Zn_2Cys_6 cluster and coiled-coil domains of AtrR (amino acids 50-160) as well as the basic region/helix-loop-helix/leucine zipper (bHLHZ) region of *A. fumigatus* SrbA (amino acids 161-267) were expressed as N-terminal maltose-binding protein (MBP) fusion separated by a tobacco etch virus (TEV) protease site in *E. coli* BL21(DE3), *via* a modified pET28a vector (Novagen; Merck KGaA, Darmstadt, Germany). Crude bacterial lysates were purified by Dextrin Sepharose affinity chromatography (Cytiva, Marlborough, Massachusetts, USA) after overnight autoinduction (Overnight Express Instant TB Medium, Novagen; Merck KGaA, Darmstadt, Germany) and cell lysis. The MBP fusion proteins were cleaved with TEV protease and further purified sequentially (Fig. S2) using Cellufine Sulfate (Merck Millipore, Burlington, Massachusetts, USA) cation exchange chromatography (CIEX), $(NH_4)_2SO_4$ precipitation (70% w/v for $AtrR_{50-160}$, 50% for $SrbA_{161-267}$). Proteins were further purified by size exclusion chromatography (SEC) on a Superdex prep grade 75 16/60 column (Cytiva, Marlborough, Massachusetts, USA) in 20 mM HEPES, 150 mM NaCl, 10 µM $Zn(OAc)_2$, pH 7.5 ($AtrR_{50-160}$) or 20 mM HEPES, 300 mM NaCl, pH 7.5 ($SrbA_{161-267}$).

References

1. Fraczek MG, Bromley M, Buied A, Moore CB, Rajendran R, Rautemaa R, Ramage G, Denning DW, Bowyer P. 2013. The *cdr1B* efflux transporter is associated with non-*cyp51a*-mediated itraconazole resistance in *Aspergillus fumigatus*. Journal of Antimicrobial Chemotherapy 68:1486-1496.
2. Gsaller F, Hortschansky P, Furukawa T, Carr PD, Rash B, Capilla J, Muller C, Bracher F, Bowyer P, Haas H, Brakhage AA, Bromley MJ. 2016. Sterol Biosynthesis and Azole Tolerance Is Governed by the Opposing Actions of SrbA and the CCAAT Binding Complex. Plos Pathogens 12.
3. Baldin C, Kuhbacher A, Merschak P, Sastre-Velasquez LE, Abt B, Dietl AM, Haas H, Gsaller F. 2021. Inducible Selectable Marker Genes to Improve *Aspergillus fumigatus* Genetic Manipulation. J Fungi (Basel) 7.
4. Guinea J, Meletiadis J, Arikan-Akdagli S, Muehlethaler K, Kahlmeter G, Arendrup MC, Eucast A. 2022. EUCAST definitive document EDef 9.4: Method for the determination of broth dilution

minimum inhibitory concentrations of antifungal agents for conidia forming moulds EDef 9.4 (EUCAST-AFST).
5. Hortschansky P, Eisendle M, Al-Abdallah Q, Schmidt AD, Bergmann S, Thon M, Kniemeyer O, Abt B, Seeber B, Werner ER, Kato M, Brakhage AA, Haas H. 2007. Interaction of HapX with the CCAAT-binding complex - a novel mechanism of gene regulation by iron. Embo Journal 26:3157-3168.
6. Furukawa T, Scheven MT, Misslinger M, Zhao C, Hoefgen S, Gsaller F, Lau J, Jochl C, Donaldson I, Valiante V, Brakhage AA, Bromley MJ, Haas H, Hortschansky P. 2020. The fungal CCAAT-binding complex and HapX display highly variable but evolutionary conserved synergetic promoter-specific DNA recognition. Nucleic Acids Res doi:10.1093/nar/gkaa109.
7. Furukawa T, van Rhijn N, Fraczek M, Gsaller F, Davies E, Carr P, Gago S, Fortune-Grant R, Rahman S, Gilsenan JM, Houlder E, Kowalski CH, Raj S, Paul S, Cook P, Parker JE, Kelly S, Cramer RA, Latge JP, Moye-Rowley S, Bignell E, Bowyer P, Bromley MJ. 2020. The negative cofactor 2 complex is a key regulator of drug resistance in *Aspergillus fumigatus*. Nature Communications 11.

Figure Legends

Fig. S1 **Schematic representation of *cyp51A* deletion and site-directed integration of plasmids containing *cyp51A* expression constructs.** A Δ*cyp51A* strain was generated using the FusionPCR approach. pcyp51A plasmids were linearized using *Kpn*I and transformed into Δ*cyp51A*.

Fig. S2 **SDS-PAGE analysis showing the sequential purification steps of recombinant *A. fumigatus* $AtrR_{50-160}$ (A) and $SrbA_{161-267}$ (B) proteins from bacterial cell lysates.** SPR protein-DNA interaction measurements of $AtrR_{50-160}$ and $SrbA_{161-267}$ were performed as described for the CBC, except the following modifications: $AtrR_{50-160}$ samples containing 100 µg/ml salmon sperm DNA (Thermo Fisher Scientific, Waltham, Massachusetts, USA) were injected in running buffer A (10 mM HEPES pH 7.4, 150 mM NaCl, 0.005% (v/v) surfactant P20, 5 mM β-Mercaptoethanol, 10 µM $ZnCl_2$) at concentrations from 1.56 to 100 nM. Sample injection and dissociation times were set to 100 and 200 seconds. Running buffer B (10 mM HEPES pH 7.4, 150 mM NaCl, 3 mM EDTA, 0.05% (v/v) surfactant P20) was used for injection of $SrbA_{161-267}$ samples containing 5 µg/ml salmon sperm DNA at concentrations from 12.5 to 200 nM. Injection and dissociation times were set to 200 and 400 seconds.

Table S1. **Strains used in this study.**

Strain	Genotype	Reference
A1160P+ (wt)	*Δku80, pyrG+*	(1)
Δ*hapX*	Δ*hapX::hph*	(2)
Δ*hapC*	Δ*hapC::hph*	(2)
Δ*atrR*	Δ*atrR::hph*	(7)
Δ*srbA*	Δ*srbA::hph*	(2)
Δ*cyp51A*	Δ*cyp51A::ble*	This study
cyp51A^{WT}	*Δcyp51A::ble; Pcyp51A*^{WT}*-cyp51A; hph*	This study
cyp51A^{HapX*}	*Δcyp51A::ble; Pcyp51A*^{HapX*}*-cyp51A; hph*	This study
cyp51A^{CBC*}	*Δcyp51A::ble; Pcyp51A*^{CBC*}*-cyp51A; hph*	This study
cyp51A^{AtrR*}	*Δcyp51A::ble; Pcyp51A*^{AtrR*}*-cyp51A; hph*	This study
cyp51A^{SrbA*}	*Δcyp51A::ble; Pcyp51A*^{SrbA*}*-cyp51A; hph*	This study
cyp51A^{AtrR*CBC*}	*Δcyp51A::ble; Pcyp51A*^{AtrR*CBC*}*-cyp51A; hph*	This study

Table S2. **Oligonucleotides used in this study.**

**Primer name**	**Sequence 5' → 3'**
Generation of pcyp51A^{WT}	
pAN 7.1 cyp51A-FW	TTCAAACTGGTGATGCGGTATTTTCTCCTTACG
pAN 7.1 cyp51A-RV	AAGATTTGTGAAGGGCGAATTCGTTTAAACCTG
cyp51A-FW	ATTCGCCCTTCACAAATCTTTGACTCATACCCCC
cyp51A-RV	TACCGCATCACCAGTTTGAACACGGAACCT
Generation of point mutations in transcription factor binding motifs	
HapX*-FW	AATGAAAGTTGCCTAATTAGCGAGGTGTAGTTCCAGCATACCATACAC
HapX*-RV	CGCTAATTAGGCAACTTTCATTCGGCTCAGCACACATCCG
CBC*-FW	ATGTGTGCTGAGCCGGCGGAAAGTTGCCTAATTACTAAGGTGTAGTTCC
CBC*-RV	CCGCCGGCTCAGCACACATCCGG
AtrR*-FW	TCTAGAATCACGCGGTCTACATGTGTGCTGAGCCGAATGAAAG
AtrR*-RV	TGTAGACCGCGTGATTCTAGACAACTCT
SrbA*-FW	GAATTCCGAAGTCCGGATGTGTGCTGAGC
SrbA*-RV	CATCCGGACTTCGGAATTCTAGACAACTCTGAAGTGGTGCTG
AtrR*CBC*-FW	TGAGCGGTATGAAAGTTGCCTAATTACTAAGGTGT
AtrR*CBC*-RV	AATTAGGCAACTTTCATACCGCTCAGCACACATCCGGAC

Table S3. **Azole susceptibility of *Pcyp51A* mutant strains under different iron conditions.** MIC levels were determined using the EUCAST broth microdilution reference method (4).

	**MIC [µg/mL]**								
	Voriconazole			Posaconazole			Itraconazole		
	-Fe	+Fe	hFe	-Fe	+Fe	hFe	-Fe	+Fe	hFe
cyp51A^{WT}	0.125	0.25	0.25	0.016-0.031	0.063	0.063	0.063	0.125	0.125
cyp51A^{HapX*}	0.25-0.5	0.5	0.5	0.063	0.125	0.125	0.125-0.25	0.25	0.25-0.5
cyp51A^{CBC*}	0.125	0.25	0.25	0.016	0.063	0.031	0.063	0.125	0.125
cyp51A^{AtrR*}	0.063	0.125	0.125	0.002	0.004	0.004	0.008	0.031	0.031
cyp51A^{SrbA*}	0.063	0.125	0.125	0.002	0.004	0.004	0.008	0.031	0.031
cyp51A^{AtrR*CBC*}	0.063	0.125	0.125	0.002	0.004	0.004	0.008	0.031	0.031

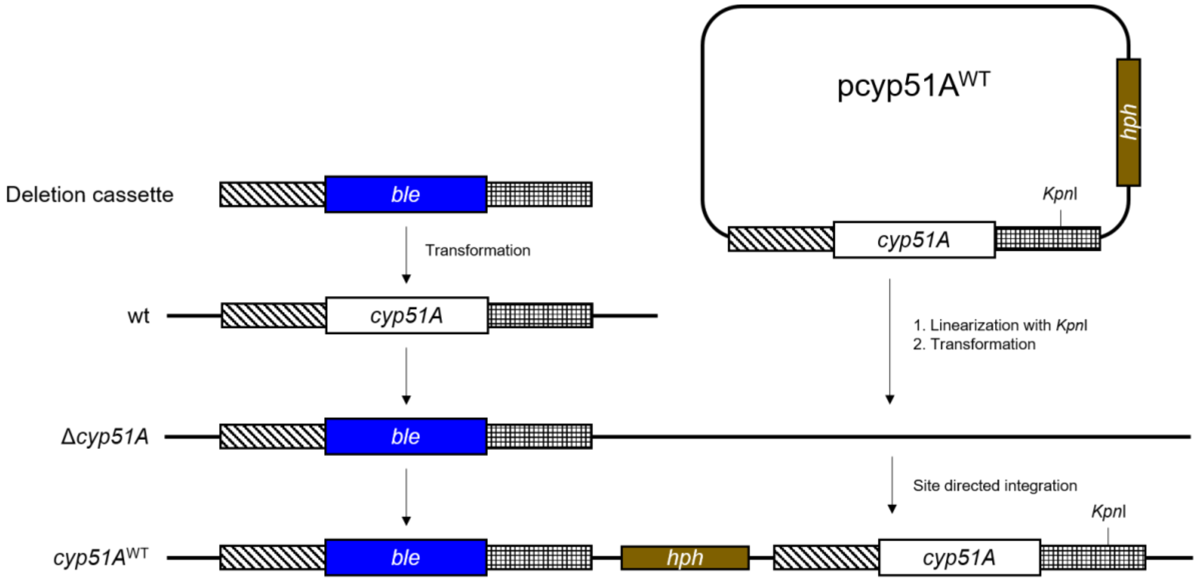
pcyp51A^WT
hph
KpnI
Deletion cassette
ble
Transformation
cyp51A
wt
1. Linearization with KpnI
2. Transformation
Δcyp51A
Site directed integration
cyp51A^WT

Fig. S1

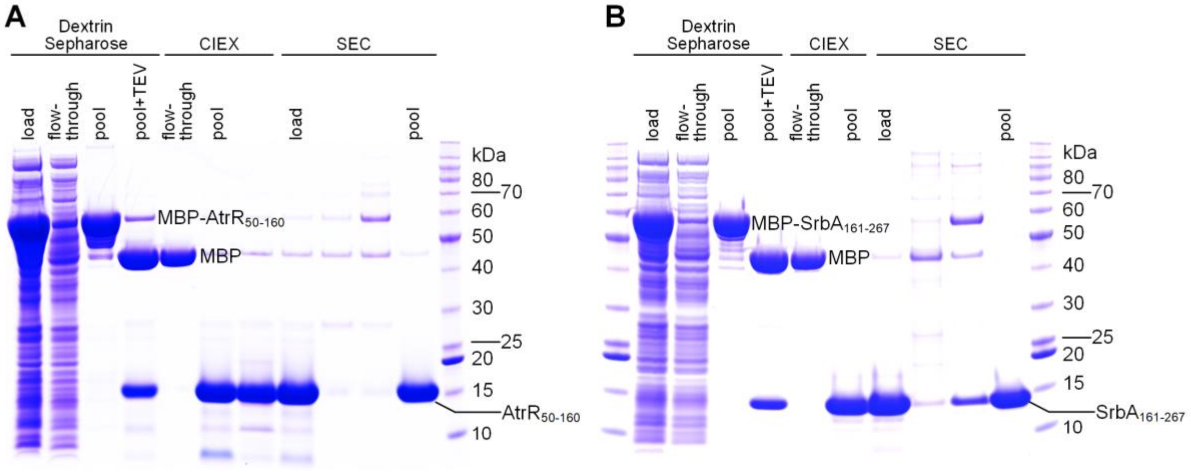
A
Dextrin Sepharose
CIEX
SEC
load
flow-through
pool
pool+TEV
flow-through
pool
load
pool
kDa
80
70
60
50
40
30
25
20
15
10
MBP-AtrR50-160
MBP
AtrR50-160
B
Dextrin Sepharose
CIEX
SEC
load
flow-through
pool
pool+TEV
flow-through
pool
load
pool
kDa
80
70
60
50
40
30
25
20
15
10
MBP-SrbA161-267
MBP
SrbA161-267

Fig. S2
